# The cost-analysis of Team-Based Learning versus small group interactive learning in undergraduate medical education

**DOI:** 10.1186/s12909-025-07844-x

**Published:** 2025-10-02

**Authors:** Irene Sterpu, Lotta Herling, Anna Möller, Helena Kopp Kallner, Hedvig Engberg, Jonas Nordquist, Ganesh Acharya

**Affiliations:** 1 Division of Obstetrics and Gynecology, Department of Clinical Sciences, Intervention and Technology (CLINTEC), Karolinska Institutet, Stockholm, Sweden; 2 Center for Fetal Medicine, Pregnancy Care and Delivery, Karolinska University Hospital, Stockholm, Sweden; 3 Department of Clinical Science and Education, Karolinska Institutet, Stockholm, Sweden; 4 Department of Obstetrics and Gynecology, Stockholm South Hospital, Stockholm, Sweden; 5 Department of Clinical Sciences Danderyd Hospital, Karolinska Institutet, Stockholm, Sweden; 6 Department of Obstetrics and Gynecology Danderyd Hospital, Stockholm, Sweden; 7 Department of Women’s and Children’s Health, Karolinska Institutet, Stockholm, Sweden; 8 Department of Gynecology and Reproductive Medicine, Karolinska University Hospital, Stockholm, Sweden; 9 Department of Medicine (Huddinge), Karolinska Institutet, Stockholm, Sweden; 10 Department of Clinical Medicine, UiT The Arctic University of Norway, Tromsø, Norway

**Keywords:** Cost-analysis, Team-based learning, Active learning, Cost-efectiveness

## Abstract

**Introduction:**

The financial resources for medical education are limited. Since every pedagogical intervention uses these resources, it is important to prioritize interventions that generate the most educational gains. Applying cost-analysis in education helps align financial resources with educational goals. Team-based learning (TBL) and small group interactive learning (SIL) are two active teaching/learning methods widely used in medical education. We have previously shown that their outcomes are comparable regarding students’ knowledge acquisition, satisfaction and engagement. The aim of this study was to evaluate the costs of delivering TBL versus SIL to undergraduate medical students.

**Method:**

The implementation costs and recurring costs of delivering TBL and SIL to 157 medical students attending clinical seminars in the obstetrics and gynecology course at Karolinska Institutet were evaluated. A cost minimization analysis was performed where the implementation costs and the recurring costs of delivering TBL and SIL were calculated and compared.

**Results:**

The implementation costs of delivering TBL were 126 USD/student, with teacher salaries accounting for 50% of the total costs. The recurring costs of delivering TBL and SIL were 74 USD/student and 31 USD/student, respectively. The sensitivity analysis for TBL showed that with analog aids and a student: teacher ratio of 80:1, the costs would be 10 USD/student.

**Conclusion:**

TBL was more costly than SIL in our setting, primarily due to the implementation expenses incurred for faculty training during the initial phase of this newly introduced teaching method and the costs of using an electronic platform. However, the sensitivity analysis indicated that adopting analog tools and increasing the student: teacher ratio could significantly reduce TBL costs, making it more comparable to SIL. Keywords: cost-analysis, team-based learning, active learning, cost-efectiveness

## Background

Medical education continuously evolves to adopt teaching methodologies that enhance learning outcomes, engagement, critical thinking, and cost-effectiveness. Among these methodologies, Team-Based Learning (TBL) is a learner-centered educational strategy, aimed to foster collaboration, discussion, and communication among students as well as peer feedback. TBL also aims to address the challenges associated with active learning and time demands on faculty [1]. By dividing a sizable group of 100 or more students, into smaller subgroups, TBL promotes active engagement among students, with the facilitation managed by a single faculty member [2]. Currently, TBL is used in various health professions´ educational settings globally including schools of medicine, nursing, dentistry, and pharmacy [3–7].

The overall cost of medical education is difficult to assess, primarily because the costs of all components are usually not considered or properly calculated, and secondly, because the effectiveness of the education is difficult to measure due to the complexity of learning outcomes, diversity of training programs, and lack of agreed criteria or standards regarding assessment methods [8].

Healthcare systems globally are encountering shortage in resources, including those allocated for medical education. Therefore, medical universities must adopt effective educational methodologies that are appropriate for their courses to ensure students’ knowledge acquisition and maximize the value returned to society. Moreover, accountability is essential when allocating public funds to medical education. Transparent cost analysis, conducted with methodological rigor, fosters trust and ensures public support for ongoing and future funding [8]. These considerations underscore the importance of performing cost evaluations to ensure the sustainability and effectiveness of medical education.

Studies on cost-effectiveness have been performed for other pedagogical methods, such as problem-based learning (PBL) [9] and interprofessional learning [10]. To our knowledge, no previous studies have evaluated the costs of implementing TBL in clinical disciplines in undergraduate medical education.

Our study aimed to evaluate and compare the costs of TBL versus traditional small group interactive learning (SIL) in undergraduate medical education, by analyzing both the financial and educational implications of each approach.

## Method

In this study, we calculated the costs of introducing and using TBL and compared with costs related to SIL, in a setting where SIL is the incumbent teaching method. The implementation costs of transiting to and implementing TBL in the obstetrics and gynecology course for undergraduate medical education at Karolinska Institutet, a medical university in Sweden, were calculated. These costs included training of the teaching faculty to get ready for TBL, and preparation and delivery of the first TBL seminar. The recurring costs of each iteration were then calculated for the TBL seminars, and these results were compared with the costs of delivering traditional SIL seminars.

### Definitions

According to the principles of health economic evaluation, a cost minimization analysis measures and compares the costs of two different interventions, and is appropriate to use when the alternatives compared are known or assumed to have the same outcomes [11].

### Context

During one semester, a total of 157 students attended the obstetrics and gynecology clinical rotation, which is part of the 5th-year curriculum. A typical 6-week clinical rotation in the specialty consists of several lectures, 5 seminars, and clinical rotation, where students participate alongside obstetricians and gynecologists in their everyday clinical work. The traditional SIL seminars consisted of a small group interactive teaching and learning exercise with discussions based on clinical scenarios, with a student to teacher ratio of 10:1. The TBL seminars had a student to teacher ratio of 20:1. Two seminars (one in obstetrics and one in gynecology) were designed and delivered in two different formats, i.e. TBL and SIL. The teachers were clinicians, with several years of teaching experience using SIL, who have additionally been trained in delivering TBL. Training in TBL involved five online sessions, each lasting four hours for designing TBL seminars, creating individual readiness assurance test (iRAT) and team readiness assurance test (tRAT) questions, application exercises, and facilitating TBL activities.

### Pedagogic outcomes for TBL and SIL

We have previously published a comparative evaluation of students’ knowledge acquisition, engagement and satisfaction with respective teaching methods. The results of pedagogic outcomes of TBL and SIL were similar without any statistically significant differences [12].

### Economic evaluation

The costs identification started with the inventory of all relevant resources used for delivering TBL and SIL according to Levin’s ingredients method [13]. Both direct costs, which incurred directly during the production or delivery of the seminars, and indirect costs which incurred indirectly, such as the administrative support, were taken into consideration. The costs included in this analysis were identified in agreement with previously published reviews regarding the most important items that should be included in the costs analysis of medical education, such as: training time, faculty time, equipment, admin/staff time, and facilities [14, 15].

The implementation costs of TBL, and recurring costs of delivering TBL and delivering SIL seminars were calculated. The implementation costs of TBL are defined as the one-time expenses associated with training teachers in the new teaching method, developing a TBL curriculum, and preparing and delivering the first TBL seminar. The recurring costs are defined as the expenses incurred during each semester for teaching the undergraduate students using either TBL or SIL. The faculty´s teaching workload was calculated by considering both the teaching time (i.e. the actual classroom teaching time, the preparation time and the time required for updating seminars), as well as the costs for training the teachers in TBL, and consisted of the following items:

1. Personnel costs: staff costs estimates were based on average salaries of consultants in obstetrics and gynecology in Stockholm County, and average salaries of administrative personnel at Karolinska Institutet in the year 2023. The time estimation for both the faculty and the administrative personnel were based on diaries of the faculty time and administrative records.
2. Costs for technical platform InteDashboard^™^ (CognaLearn Pte.Ltd) are paid per student per year, which means that the costs are not solely supported by one course. We chose to integrate those costs since our course was the only one using this platform for the 5th-year students at Karolinska Institutet.
3. Overhead costs, such as the costs of TBL training program organized by Karolinska Institutet, were not calculated since the training program was part of a bigger initiative. Karolinska Institutet has been sequentially implementing TBL as a pedagogical method in the 6-year medical school curriculum since 2021 and created its own internal TBL training program for teachers. Therefore, there were no additional costs for the TBL training of our teaching faculty besides the teachers’ salaries for the time they spent in these training courses, which is accounted for.
4. At Karolinska Institutet, room costs are determined based on several factors, including room size (defined by the number of students that can be comfortably accommodated), day of the week (Saturday and Sunday costing more than Monday to Friday bookings), time of the day (higher cost between 9:00–12:00 h) and whether the room is equipped with computers or not. In our specific context, rooms for SIL were provided at no cost at the teaching hospital. However, we acknowledge that this may not be the case in other institutions and therefore incorporated a hypothetical room cost for SIL based on Karolinska Institutet’s costing criteria for room renting.

The currency conversion was 1 Swedish crown = 1 USD10,23 September 2024 (market exchange rate Sveriges Riksbank).

The study analyses the costs of delivering seminars from the perspective of clinical obstetrics and gynecology course in a single institution.

### Sensitivity analysis

A deterministic sensitivity analysis was performed [16]. The plausible scenarios tested for the implementation phase of TBL, included following conditions: increasing the ratio of student: teachers to 40:1 and 80:1, the use of traditional lecture theaters and the use of analog aids instead of Intedashboard in the seminars.

The study protocol was reviewed by the Swedish Ethical Review Authority and granted exempt status on 2022.06.22 (Ref. Dnr: 2022-02891-01). All students participating in the study provided written informed consent.

## Results

### Estimated costs of delivering TBL and SIL

#### Implementation costs

The implementation costs of delivering TBL consisted mainly of the teachers’ time for training in the new methodology and time required to create and develop the material for the TBL seminars (Table 1). There were no additional costs for providing the students with computers since each student came to the seminars (both TBL and SIL) with their own computer or smartphone.

**Table 1:** Implementation costs of implementing and delivering TBL in the obstetrics and gynecology course for undergraduate medical students

	Hours or students	Costs/h or Costs/student (USD)^#^	Total costs (USD)
Training for teachers (*n* = 7)	140 h	53	7420
Developing the seminars	16 h	53	848
Teaching time	32 h	53	1696
Administrators training in TBL/Intedashboard	16 h	22	352
Administrative help with the seminars	12 h	22	264
Costs of renting classrooms			2145
Administrative costs for Intedashboard (optional costs)	157 students	45	7065
Total			19,790

^#^All costs include all relevant taxes

The implementation cost was 126 USD/student with teacher salaries accounting for 50% of the total cost.

The recurring costs of TBL and SIL are presented in Tables 2 and 3, respectively. The main recurring costs of delivering SIL were the costs associated with teaching whilst the costs for TBL are associated with the use of digital aid. The recurring costs of delivering TBL in a clinical rotation was 74 USD/student when using an electronic platform. This cost could be reduced to 29 USD/student if analog aids were used. In comparison, the recurring cost of delivering SIL was 31 USD/student.

**Table 2:** Recurring costs of delivering TBL in obstetrics and gynecology to undergraduate medical students

	Hours or student	Costs/h or Costs/student (USD)	Total costs (USD)
Developing the seminars	8 h	53	424
Teaching time	32 h	53	1696
Administrative help with the seminars	12 h	22	264
Costs of renting classrooms	32 h		2145
Total			4529
Administrative costs for Intedashboard (optional)	157 students	45	7065
Grand Total (with optional costs)			11,594

The costs for the technical platform (InteDashboard™) used in TBL constituted 36% of the total costs in the implementation phase and 61% in the recurrent phase.

**Table 3:** Recurring costs for delivering SIL in obstetrics and gynecology to undergraduate medical students

	Hours	Costs/h (USD)	Total costs (USD)
Developing the seminars	16 h	53	848
Teaching time	64 h	53	3392
Administrative help with the seminars	8 h	22	176
Costs of renting classrooms	64 h		510
Total			4926

The sensitivity analysis was performed according to the scenarios described in the methods and are presented in Table 4 for the implementation phase and in Table 5 for the recurring phase. The costs of implementation of TBL decreased considerably with the use of analog aids and increasing the student: teacher ratio.

**Table 4:** Sensitivity analysis for the implementation phase of TBL

Scenario	Total implementation costs TBL (USD)	Costs/student (USD)
1. Increasing student: teacher ratio to 40:1	17,782	113
2. Increasing student: teacher ratio to 80:1	16,821	107
3. Analog aids instead of Intedashboard	12,725	81
4. Analog aids (student: teacher ratio 80:1)	9756	62

**Table 5:** Sensitivity analysis for the recurring phase TBL

Scenario	Total implementation costs TBL (USD)	Costs/student (USD)
1. Increasing student: teacher ratio to 40:1	9585	61
2. Increasing student: teachers ratio to 80:1	8619	55
3. Analog aids instead of Intedashboard	4529	29
4. Analog aids (student: teacher ratio 80:1)	1554	10

## Discussion

We found that the cost of TBL is higher compared to SIL and the higher initial cost of delivering TBL in the implementation phase was mainly due to the time required to train teaching personnel in this method. However, it implies that teachers were already experienced in SIL and do not require additional training in this traditional teaching method. The recurring costs of delivering TBL were still higher than SIL and mainly consisted of the costs for digital support. The sensitivity analysis for the recurring phase of delivering TBL showed that the use of analog aids and increasing the student-to-teacher ratio would decrease the costs of TBL considerably. Increasing the student-to-teacher ratio up to 100:1 without compromising learning outcomes has been reported [2, 17].

While a substantial part of the faculty time in TBL was allocated to developing materials for the TBL sessions, in SIL most of the faculty time was allocated to classroom teaching. Faculty development is important during the implementation of any new teaching method. A previous study has shown that a successful implementation of TBL often requires training the faculty in the method and facilitation skills [18]. However, regular skills training and appraisals could improve the quality of SIL too.

Levin et al. suggest that cost-effectiveness should be a cornerstone of educational policy. Many high-cost interventions may not provide proportional benefits compared to less expensive alternatives. For instance, investing in teacher training and professional development often yields better student outcomes than reducing class sizes, and are also more affordable [19].

Rajalingam et al. [20] and Espey et al. [21] previously discussed that team-centric learning spaces are important in TBL, and students’ perspectives on TBL improve when they feel more comfortable with the physical space and find it easy to communicate with each other. Many institutions, including ours, have migrated to a TBL curriculum while the learning spaces are still mostly conventional lecture theaters and seminar rooms. Our teaching faculty decided that the additional costs for TBL rooms were not justified and continued delivering TBL seminars in our traditional learning lecture theaters. Further investigation is needed to understand how this affects the success of TBL sessions and the satisfaction of both students and faculty. In this study there were no differences in students’ satisfaction between TBL and SIL as reported previously [12], but we have not evaluated faculty preferences.

In resource constrained healthcare systems there is a need for efficient allocation of resources, including faculty, facilities, and technology. This involves optimizing class sizes, student-to-teacher ratios, and ensuring that resources are directed towards initiatives that contribute to learning outcomes.

Overall, while TBL may initially appear more expensive, strategic implementation and cost mitigation strategies, such as increasing class sizes, use of analog aids, implementing TBL across multiple disciplines and in that way spreading the costs of license fees for electronic platforms, could make it a more sustainable option in the long run. TBL can be conducted using analog methods without a digital dashboard. In fact, Michelsson, the original developer of the method, still employs scratch cards and other non-digital tools in TBL sessions. While Intedashboard provides certain advantages, such as streamlining administration, enhancing real-time feedback, and facilitating data collection, it is not an essential requirement for conducting TBL.

An important consideration in interpreting our findings is the concept of willingness to pay (WTP) for educational innovations. While TBL may incur higher upfront and recurring costs compared to SIL, institutions may deem these additional expenses acceptable if they are associated with qualitative benefits that are difficult to capture in traditional cost-effectiveness analyses. The choice between TBL and SIL should consider not only direct financial costs, but also educational benefits associated with each method. Differences in institutional infrastructure, faculty salaries, and educational culture could also influence transferability. TBL’s structured approach to fostering teamwork, communication, problem-solving, and application of knowledge in clinical contexts offers distinct benefits. The willingness to pay for these benefits will likely vary across institutions depending on available resources, educational priorities, and the extent to which such competencies are considered essential learning outcomes. Including WTP in future economic evaluations could help stakeholders make informed decisions about the adoption and scaling of TBL in different contexts.

An important limitation when interpreting results of cost-effectiveness in medical education is the lack of established benchmarks for cost-effectiveness analogous to cost per QALY in healthcare, which makes it challenging to determine what constitutes a “reasonable” cost for achieving a given educational outcome. Developing such standards could support more informed decision-making and priority setting in educational investments.

To our knowledge, this is the first study analyzing the implementation and recurring costs of TBL in a clinical discipline for undergraduate medical students. The framework can be applied and adapted for different contexts. One of the limitations is that not all indirect costs could be calculated, such as lighting, heating, and maintenance of tutorial rooms, since these costs in our setting are partially covered by the hospitals. Another limitation is that we only used a student-to-teacher ratio of 20:1 in TBL, but according to the TBL methodology, this can be increased further [2] which can be expected to further reduce the costs. It should also be taken into consideration that the students had their own computers/smartphones for the seminars since this is a standard practice in our medical university, which considerably decreased the costs of delivering the seminars. However, this applies to both TBL and SIL. An indirect gain from the TBL seminars is the clinical or research time (32 h) that could be freed for teachers, as all of them work as clinicians and researchers.

Generalizability of our findings could be questioned since certain components of cost (e.g. faculty time, administrative costs, software expenses) may vary across institutions and countries. However, the general principles of cost calculation and the potential for cost reduction through increased student-to-teacher ratios and use of analog tools remain broadly applicable. Our approach of reporting cost per student, while intuitive and commonly used in educational cost analyses, does not fully account for economies of scale. In reality, the marginal cost of adding an additional student is likely to decrease once the fixed costs (e.g. faculty training, room bookings, faculty teaching time) are covered. Future research should explore alternative analytical frameworks that better capture such cost dynamics.

There are numerous studies showing the learning benefits of TBL in comparison with traditional lectures in preclinical and clinical disciplines [22–26] with these two methods having similar student-to-teacher ratios. However, there are no studies on implementation and recurring costs of implementing and delivering TBL. Future studies should expand these insights by conducting direct comparisons of TBL and SIL within similar institutional settings, considering factors such as student outcomes, faculty workload, and long-term financial sustainability. Such analyses will be instrumental in guiding medical schools towards cost-effective and educationally robust teaching strategies. Comprehensive cost analyses can also guide educational and policy decisions and help make informed decisions about future investments in medical education.

## Conclusion

In our setting, TBL was associated with high implementation costs, primarily due to faculty training expenses during the implementation phase. Additionally, the recurring costs for TBL delivery were elevated due to the use of technological platforms, such as InteDashboard™. However, sensitivity analysis indicated that adopting analog tools and increasing the student-to-teacher ratio could significantly reduce TBL costs, making it more comparable to SIL. These findings highlight key cost considerations for institutions implementing TBL and suggest potential strategies to optimize its cost-effectiveness.

By providing a cost analysis, this research contributes to the ongoing discourse on optimizing teaching methodologies in medical education, balancing both pedagogical effectiveness as well as financial sustainability.

## Data Availability

Data will be available on request to the corresponding author irene.sterpu@ki.se.
